# High Expression of MicroRNA-196a Indicates Poor Prognosis in Resected Pancreatic Neuroendocrine Tumor

**DOI:** 10.1097/MD.0000000000002224

**Published:** 2015-12-18

**Authors:** Yoon Suk Lee, Haeryoung Kim, Hyoung Woo Kim, Jong-Chan Lee, Kyu-Hyun Paik, Jingu Kang, Jaihwan Kim, Yoo-Seok Yoon, Ho-Seong Han, Insuk Sohn, Jeonghee Cho, Jin-Hyeok Hwang

**Affiliations:** From the Department of Internal Medicine (YSL, HWK, JCL, KHP, JK, JK, JHH); Department of Pathology (HK); Department of Surgery (YSY, HSH), Seoul National University College of Medicine, Seoul National University Bundang Hospital, Seongnam; Department of Internal Medicine, Keimyung University School of Medicine, Dongsan Medical Center, Daegu (YSL); Biostatistics and Clinical Epidemiology Center, Samsung Medical Center (IS); and Samsung Genome Institute, Samsung Medical Center, Seoul; Department of Nanobiomedical Science, Dankook University, Cheonan (JC).

## Abstract

There is limited data on miRNA expression in pancreatic neuroendocrine tumors (PanNETs). In this study, we aimed to identify miRNAs that could be potential prognostic biomarkers of PanNETs in patients who underwent curative surgery.

For miRNA target screening, 2 primary PanNETs and corresponding liver metastases were screened for miRNA expression by the NanoString nCounter analysis. Candidate miRNAs were selected by ≥2-fold difference of expression between metastatic versus primary tumor. For miRNA target validation, quantitative real-time PCR was performed for candidate miRNAs on 37 PanNETs and matched nonneoplastic pancreata, and the miRNA levels were correlated with the clinicopathological features and patient survival data.

Eight miRNAs (miRNA-27b, -122, -142–5p, -196a, -223, -590–5p, -630, and -944) were selected as candidate miRNAs. Only miR-196a level was significantly associated with stage, and mitotic count. When PanNETs were stratified into high (n = 10) and low (n = 27) miRNA-196a expression groups, miRNA-196a-high PanNETs were significantly associated with advanced pathologic T stage (50.0% vs 7.4%), N stage (50.0% vs 3.7%), higher mitotic counts (60.0% vs 3.7%), and higher Ki-67-labeling indices (60.0% vs 22.2%). In addition, high miRNA-196a expression was significantly associated with decreased overall survival (*P* = 0.046) and disease-free survival (*P* < 0.001) during a median follow-up of 37.9 months with the hazard ratio for recurrence of 16.267 (95% confidence interval = 1.732–153.789; *P* = 0.015).

MiRNA-196a level may be a promising prognostic marker of recurrence in resected PanNETs, although further experimental investigation would be required.

## Abstract 2

Supplemental Digital Content is available in the text

## INTRODUCTION

Pancreatic neuroendocrine tumors (PanNETs) account for 1% to 2% of all pancreatic neoplasm.^1,2^ Recently, these tumors have been gaining more attention, with a 2- to 3-fold increase in incidence over the past decades.^3,4^ Complete resection is generally recommended due to their malignant potential; however, it is still debatable whether incidentally discovered small sporadic nonfunctioning PanNETs should be surgically removed. Although complete resection provides significant benefits on survival,^5^ the rate of recurrence is reported to be from 3.8% to 38.5%.^6^

MicroRNAs (miRNAs), composed of about 22 nucleotides, are receiving increasing attention as they have been shown to serve critical roles in cell proliferation, apoptosis, and developmental timing by post-transcriptional processing of their target mRNA.^7^ In addition, there is accumulating evidence that aberrant expression of miRNAs is associated with tumor prognosis and response to therapy.^8,9^ Thus, research on miRNAs has been extended to a wide variety of diseases, including solid cancers, leukemias, and autoimmune diseases such as systemic lupus erythematosus and rheumatoid arthritis.^10–14^ However, there is little data on the miRNA expression profile for PanNET: only 1 recent study has suggested that overexpression of miRNA-21 is associated with both increased proliferative activity and presence of liver metastasis.^15^ In this study, we aimed to find new miRNAs that could be potentially used as prognostic biomarkers for PanNET patients who underwent curative surgery.

## MATERIAL AND METHODS

### Patients and Study Design

A total of 43 PanNETs which were surgically resected at Seoul National University Bundang Hospital between March 2003 and July 2013 were reviewed. After excluding 6 cases (5 had liver metastasis at diagnosis and 1 case had no tissue block available), a total of 37 PanNETs were enrolled in the study. This study was designed in 2 steps: screening and validation. For miRNA target screening, we selected 2 additional patients who underwent simultaneous pancreatectomy and metastatectomy for PanNET with liver metastasis, and compared the miRNA expression profiles of the PanNETs and their matched liver metastases by the NanoString nCounter analysis. MiRNAs with ≥2-fold difference of expression between the primary and metastatic tumors were selected as potential candidate miRNAs. For miRNA target validation, quantitative real-time polymerase chain reaction (qRT-PCR) for candidate miRNAs was performed on 37 resected PanNET and matched nonneoplastic pancreata. Locked nucleic acid-fluorescent in situ hybridization (LNA-FISH) was performed for 22 cases from the same cohort with available residual tissue for further validation. All patients were followed up for tumor recurrence at regular intervals and underwent chest X-ray and abdominal computerized tomography. Disease-free survival (DFS) was defined as the interval from the date of operation to the date of tumor recurrence confirmed by imaging. Overall survival (OS) was calculated from the date of operation to the date of death or last date of follow-up. The study was approved by the human subjects committee of Seoul National University Bundang Hospital, and it followed the ethical guidelines of the 1975 Declaration of Helsinki.

### Nanostring nCounter Analysis and qRT-PCR

For the nCounter analysis, ten consecutive 8 μm-thick tissue sections from archival formalin-fixed and paraffin-embedded (FFPE) tissue from 2 primary PanNET cases and matched liver metastases were obtained, and the tumors were macrodissected using the hematoxylin-eosin-stained sections as guide slides. Total miRNA was isolated from the macrodissected tissues using the Qiagen miRNeasy Kit (Qiagen, Valencia, CA) according to the manufacturer's protocol. Total miRNA samples were analyzed for the nCounter Human miRNA Expression Assay kit (NanoString, Seattle, WA) according to manufacturer's instructions. Briefly, 100 ng of each total miRNA sample was incubated in the presence of miRNA-specific capture and reporter probes, and nonhybridized probes were removed followed by immobilization of the purified hybridized complexes. Subsequently, abundances of specific target molecules were quantified on the nCounter Digital Analyzer by counting the individual fluorescent barcodes and assessing the target molecules as previously described.^16^

Reverse transcription reactions for selected miRNAs were performed on the 37 PanNETs and corresponding nonneoplastic pancreata. Briefly, 10 consecutive 8 μm-thick tissue sections were obtained from representative archival FFPE tissue blocks from 37 PanNET cases, and the tumors and nonneoplastic pancreatic tissues were macrodissected. miRNA extraction was performed from the macrodissected tissues using the Qiagen miRNeasy Kit (Qiagen), and qRT-PCR was performed using the TaqMan miRNA Reverse Transcription Kit (Applied Biosystems, Foster City, CA) and the Applied Biosystems 7500 Real-Time PCR system (Applied Biosystems) in a total reaction volume of 15 μL. Cycle threshold (Ct) values were calculated by using the same threshold cutoff values for each assay to prevent plate-to-plate variations while analyzing data with the SDS 1.4 software (Applied Biosystems). All samples were analyzed in duplicate to confirm reproducibility. U6 snRNA was used as an internal control to normalize miRNA expression in cells. The 2^–ΔCt^ method was applied to measure the values of miRNA expression of interest. ΔCt indicates differences between Ct values of miRNAs of interest and Ct values of U6 snRNA internal control (ΔCt = CtmiRNA [sample] − CtU6sn). Then, the expression of miRNA in PanNET tissues was compared to adjacent normal pancreatic tissues by setting the value of miRNA expression in normal tissues to 1 and determining the fold change in expression against this value using the following formula: 2ΔΔCt.

### LNA-FISH for miRNA-196a

We conducted LNA-FISH for miRNA-196a on 22 PanNETs and matched nonneoplastic pancreatic parenchyma, from the same FFPE blocks as those used for the qRT-PCR analysis, as previously described.^17,18^ Hybridization was performed using an LNA oligonucleotide probe against miRNA-196a (Exiqon Inc., Woburn, MA; 1:1000) at 48 °C. U6 probes were used as positive controls. The fluorescent signals were quantified by counting the number of signals per cell by a pathologist (HK). In addition to the tumor, the same analysis was performed for acinar cells and endocrine cells (islets of Langerhans) in the nonneoplastic tissue for each case. We then calculated the ratio of signals (PanNET/acinar cells and PanNET/endocrine cells) for each case.

### Statistical Analyses

The miRNA expression levels were compared according to each clinicopathological parameter using the Mann–Whitney *U* test. The miRNA levels obtained by qRT-PCR and LNA-FISH were correlated by the Spearman correlation test. Receiver operating characteristic analysis was performed to determine the diagnostic performance of specific miRNA expression levels in identifying patients with recurrence, and high/low expression group of miRNA was determined by the optimal cutoff values. The differences between the 2 groups were compared using an independent *t*-test or Mann–Whitney *U* test for the continuous variables, and Chi-square test or Fisher exact test for the categorical variables. Survival analyses were performed using the Kaplan–Meier method and the log-rank test. Cox proportional hazard regression analyses were used to estimate hazard rations (HRs) of recurrence according to tissue miRNA levels. All potential prognostic factors with significance in univariable analysis were entered into multivariable Cox models. The final models were determined by backward elimination. All *P* values are 2-sided; *P* < 0.05 was considered statistically significant. Statistical analyses were carried out using IBM SPSS statistics version 20.0 (SPSS Inc., Chicago, IL) and STATA version 14.0 (StataCorp, College Station, TX).

## RESULTS

### Screening of Candidate miRNAs for PanNETs

**FIGURE 1:**
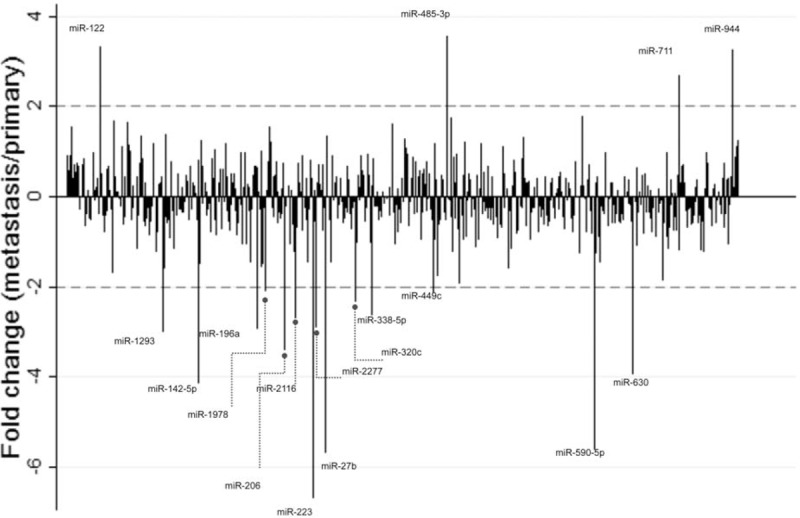
miRNA screening results in the matched primary and metastatic PanNETs. The bars demonstrate the average fold difference of expression of 654 miRNAs between primary and metastatic tumors. miRNAs demonstrating ≥2-fold difference in expression were selected for further analysis. miRNA = microRNA, PanNET = pancreatic neuroendocrine tumor.

Screening analysis of miRNAs in primary PanNETs and liver metastases by the Nanostring nCounter analysis revealed 18 miRNA candidates that were differentially expressed in both cases. miRNA-122, -485–3p, -711, and -944 were significantly 2-fold or more elevated in the metastatic tumors compared to the primary PanNETs, while miRNA −27b, −142–5p, −196a, −206, −223, −320c, −338–5p, −449c, −590–5p, −630, −1293, −1978, −2116, and −2277 were significantly 2-fold or more decreased in the liver metastases compared to primary tumor tissues (Fig. 1). From these 18 miRNA candidates, miRNA−27b, −122, −142–5p, −196a, −223, −590–5p, −630, and −944, were selected for further analysis.

### Validation of Candidate miRNAs as Prognostic Biomarkers for PanNETs

The clinicopathological characteristics of the 37 patients are summarized in Table 1. The mean age of the 37 patients was 57.2 ± 13.3 years and 54.1% of the patients were female. Nonfunctioning PanNETs comprised the majority (75.7%) of cases, and most cases (91.9%) were sporadic, while 3 cases were associated with type 1 multiple endocrine neoplasia (MEN) syndrome. PanNETs were classified as G1, G2, and G3 in 26 (70.3%), 9 (24.3%), and 2 (5.4%) cases, respectively, according to the World Health Organization (WHO) 2010 classification. During a median follow-up period of 37.9 months, recurrence occurred in 6 (16.2%) out of 37 patients.

**TABLE 1:**
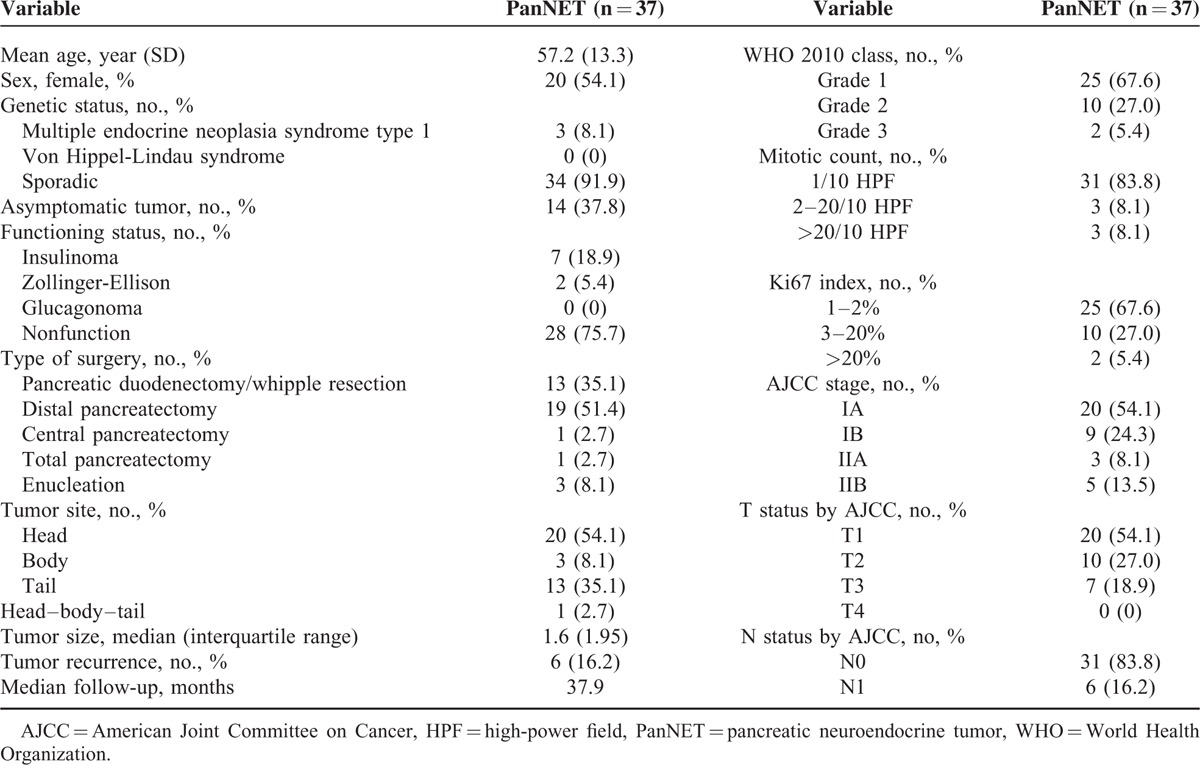
Clinicopathological Features of Pancreatic Neuroendocrine Tumors

**FIGURE 2:**
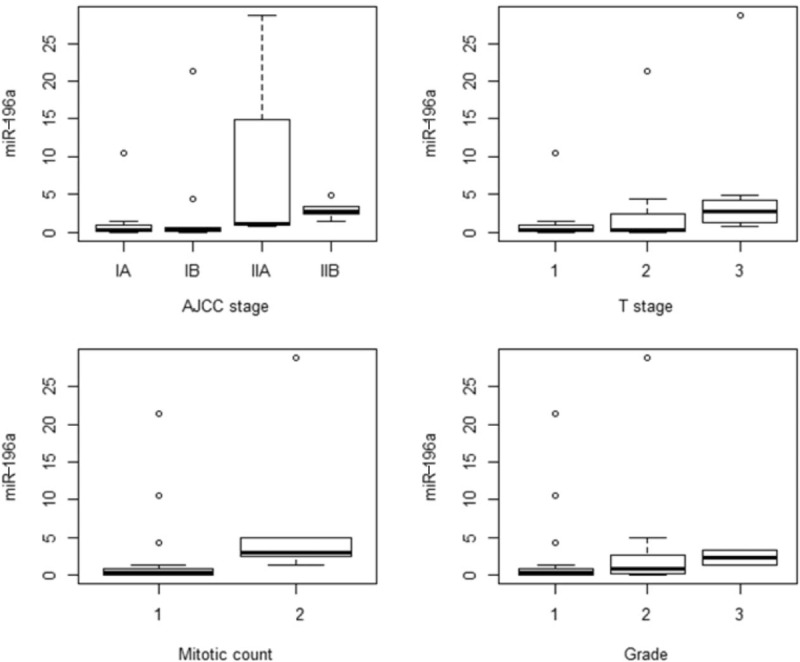
Box plots demonstrating the relationships between the clinicopathological variables and miRNA-196a levels. Higher miRNA-196a levels were seen in PanNETs with increasing AJCC stage (A), T stage (B), mitotic count (C), and WHO grade (D). AJCC = American Joint Committee on Cancer, miRNA = microRNA, PanNET = pancreatic neuroendocrine tumor, WHO = World Health Organization.

The expression levels of 8 candidate miRNAs were analyzed by qRT-PCR in the 37 PanNETs and matched nonneoplastic pancreata, and correlated with the clinicopathological factors. The expression levels of miRNA-196a were elevated in patients with pathological stage pT3 or higher (*P* = 0.004), lymph node metastasis (*P* = 0.080), American Joint Committee on Cancer stage II or higher (*P* = 0.002), high mitotic index (≥2 per 10 high-power fields [HPFs]) (*P* = 0.012), high Ki-67 labeling index (≥3%) (*P* = 0.108), and recurrence (*P* = 0.011) (Table 2, Fig. 2). MiRNA-142–5p expression was also significantly elevated in PanNETs with high mitotic index (*P* = 0.040). miRNA-27b was higher in PanNETs with high mitotic index (*P* = 0.092) and in those that recurred (*P* = 0.058), although statistical significance was not reached (Table 2).

**TABLE 2:**
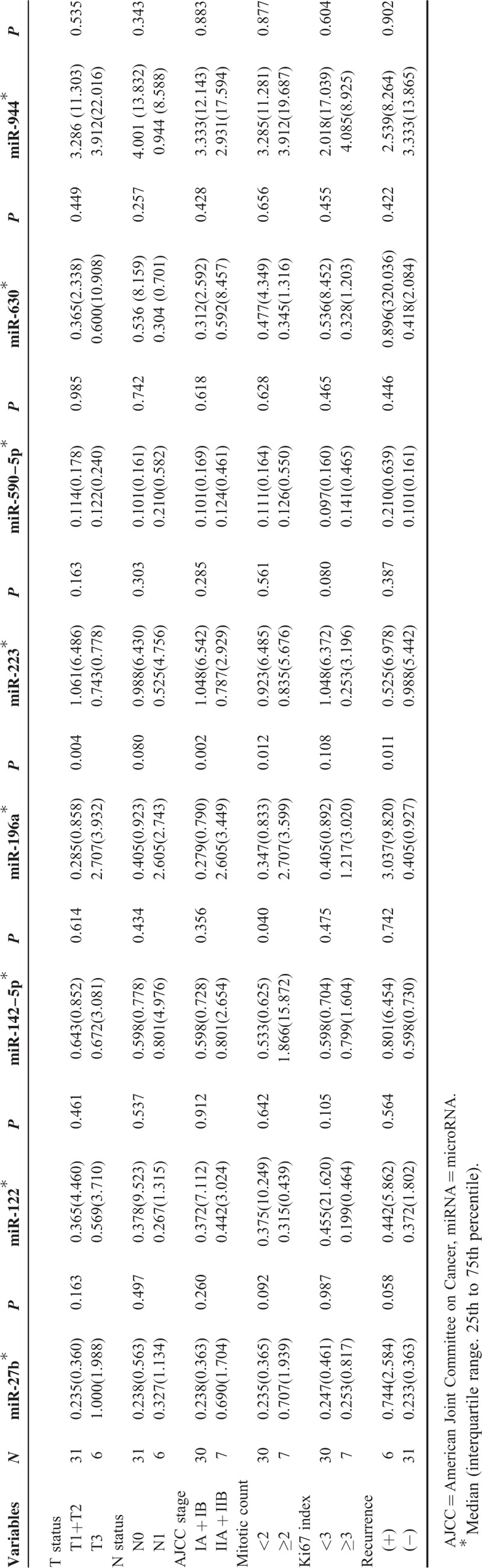
Comparison of Clinicopathological Factors and Expression Levels of miRNAs Tested

To explore the source of the increased miRNA-196a in the PanNETs in this study, we performed LNA-FISH for miRNA-196a in 22 PanNETs and matched nonneoplastic pancreata from the same cohort of cases. As acinar cells comprise the majority of the parenchymal volume, it is possible that the miRNA levels obtained by qRT-PCR from macrodissected nonneoplastic pancreata reflect the miRNA levels in the acinar cells, while PanNETs are considered to be neoplastic counterparts of endocrine cells. Therefore, we examined the miRNA-196a levels in both the acinar cells and endocrine cells (islets of Langerhans) using an in situ technique (LNA-FISH) in consecutive tissue sections from the same tissues that were used for the qRT-PCR analysis. We found that miRNA-196a signals were more abundant in PanNETs compared to corresponding nonneoplastic endocrine and acinar cells (Supplementary Figure S1), and there was a significant positive correlation between LNA-FISH and qRT-PCR results for miRNA-196a expression (*r* = 0.636, *P* = 0.003 for tumor:endocrine cell ratio; *r* = 0.636, *P* = 0.002) for tumor:acinar cell ratio.

### MiRNA-196a as a Prognostic Biomarker of PanNET

Receiver operating characteristic analyses were performed to evaluate the potential of miRNA-196a, miRNA-27b, and miRNA-142–5p as a prognostic biomarker for predicting recurrence of resected PanNET. Tissue miRNA-196a levels robustly discriminated the PanNET patients with recurrence from those without recurrence with an area under the curve (AUC) value of 0.833 (95% confidence interval [CI] = 0.658–1.000, *P* = 0.011). Using a cutoff value of relative expression 1.279 of miRNA-196a, the sensitivity and specificity for recurrence were 83.3% and 83.9%, respectively (Supplementary Figure S2). Tissue miRNA-27b levels also discriminated recurrent from nonrecurrent PanNETs with an AUC value of 0.747 (95% CI = 0.516–0.978, *P* = 0.058) using a cutoff value of 0.378, although marginally significant. No significant value was seen for miRNA-142–5p as a discriminative marker for recurrent PanNETs in this study (AUC = 0.543, 95% CI = 0.227–0.859, *P* = 0.742).

**FIGURE 3:**
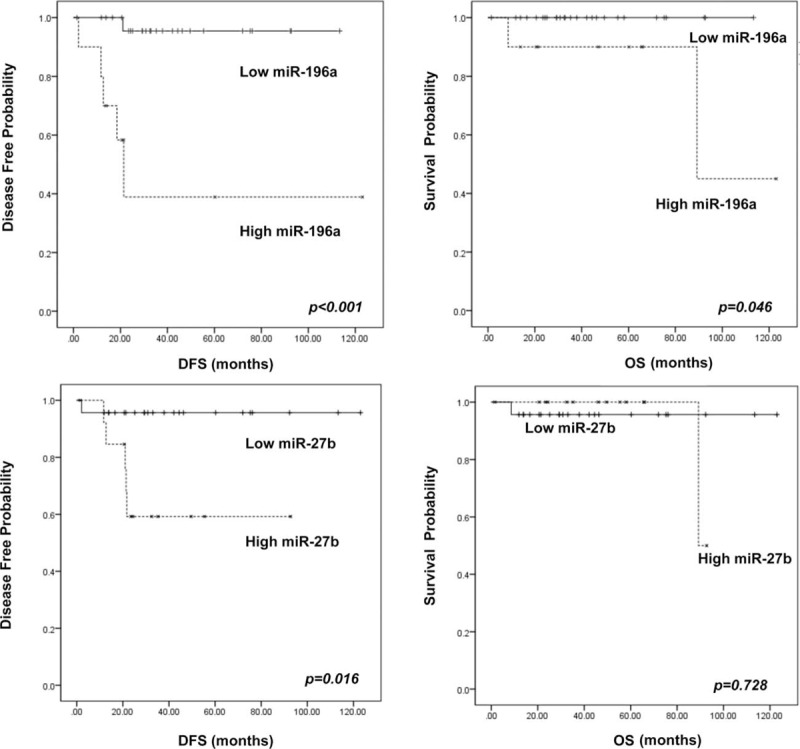
Survival analysis results. High miRNA-196a levels were associated with significantly decreased disease-free (A) and overall survivals (B). Decreased disease-free survivals were seen for PanNETs with high miRNA-27b levels (C); however, overall survival was not significantly different according to miRNA-27b status (D). miRNA = microRNA, PanNET = pancreatic neuroendocrine tumor.

The patients were dichotomized into high (n = 10) and low (n = 27) miRNA-196a expression groups, using the cutoff level of 1.279. The histological grade, mitotic index, Ki67 labeling index, frequency of angiolymphatic invasion, T stage, and N stage were significantly higher in the high miRNA-196a expression group compared to the low miRNA-196a expression group (*P* < 0.05, all) (Table 3). In addition, the high miRNA-196a group demonstrated significantly lower DFS and OS rates compared to the low miRNA-196a expression group (DFS: 38.9% vs 95.5%, *P* < 0.001; OS: 90.0% vs 100%, *P* = 0.046, respectively) (Fig. 3). Although high miRNA-27b levels in PanNETs were associated with decreased OS (*P* = 0.016), DFS was not significantly different between high and low miRNA-27b groups (Fig. 3). No significant differences in OS or DFS were seen according to miRNA-142–5p expression status. Cox regression analysis demonstrated an increased HR for recurrence in the high miRNA-196a group (HR 20.299 in univariable and HR 16.267 in multivariable analysis) (Supplementary Table S1). High miR-27b group PanNETs were also associated with increased recurrence in both univariable and multivariable analyses (HR 7.637 and 6.697, respectively), although not statistically significant.

**TABLE 3:**
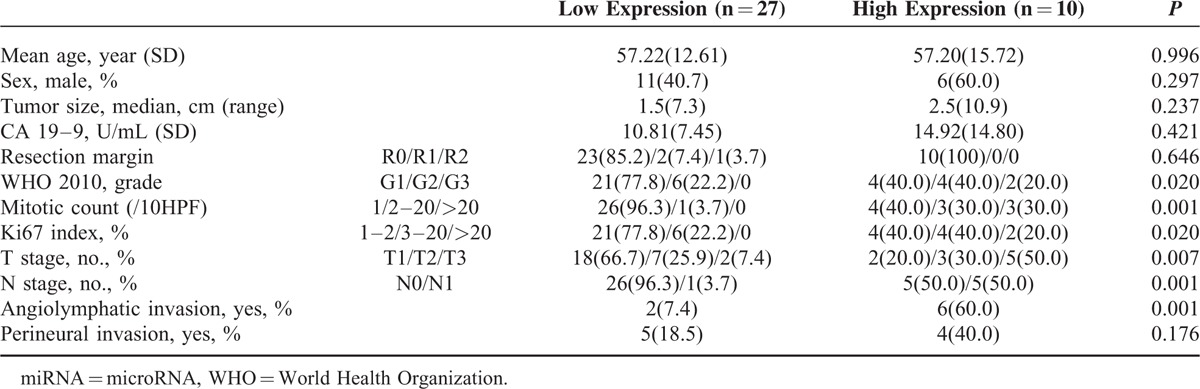
Clinicopathologic Features According to miRNA-196a Expression

As expected, WHO grade and American Joint Committee on Cancer stage were significant predictors of DFS and OS: reduced DFS was seen with increasing grade (*P* < 0.001) and stage (*P* < 0.001), and OS was also reduced with increased grade (*P* < 0.001) and stage (*P* = 0.020). In addition, the presence of lymphovascular invasion (*P* < 0.001) and perineural invasion (*P* = 0.003) were also significantly associated with decreased DFS, while OS was not significantly different according to these parameters. Interestingly, when we performed the survival analysis after stratifying for WHO grade, we found that within the group of patients with G2 PanNETs, high miRNA-196a levels were associated with lower DFS compared to G2 PanNETs with low miRNA-196a levels (*P* = 0.006).

## DISCUSSION

With the exception of pancreatic neuroendocrine microadenomas, which are defined as nonsyndromic PanNETs measuring less than 0.5 cm, all PanNETs are now regarded as malignant neoplasms under the 2010 WHO classification.^1^ However, other than tumor grade and stage, only a few prognostic biomarkers have been reported for PanNETs, and the development of novel prognostic biomarkers would be useful for the prognostic stratification of these clinically and biologically heterogeneous tumors. In this study, we demonstrate that tissue miRNA-196a expression level may be a useful biomarker for predicting recurrence and survival in PanNET patients.

During the past several years, there has been increasing interest in epigenetics research, and miRNAs have been shown to be attractive biomarkers for various malignancies, as they are stable, relatively easy to detect in serum/plasma samples and fresh and FFPE tissues, and also can be targeted therapeutically. In pancreatic ductal adenocarcinomas, the most common neoplasms occurring in the pancreas, up- or downregulation of various miRNAs, including miRNA-21, −34, −146a, −155, −196a-2, −200a/b, and −1290, have been reported.^19,20^ However, there are only a few studies on the role of miRNAs for the diagnosis and prognostication of PanNETs.

So far, studies focused on PanNETs have revealed overexpression of miRNA-21, miRNA-642 and miRNA-193b to be associated with increased proliferative activity and metastasis in PanNETs.^15,21^ We demonstrate herein that increased expression of miRNA-196a is correlated with increased tumor grade (i.e. proliferative activity demonstrated by Ki-67 and mitotic indices), higher tumor stage, and other features of aggressive behavior such as lymphovascular invasion and lymph node metastasis in PanNETs. In addition, the 5-year DFS and OS were significantly lower in patients of the high miRNA-196a expression group, suggesting a potential role of miRNA-196a as a prognostic biomarker for PanNETs after curative resection. Furthermore, although we have a limited number of cases, the presence of high miRNA-196a expression was predictive of a poor DFS in patients with G2 PanNETs, suggesting that miRNA-196a levels may provide valuable prognostic information, in addition to the current WHO grading scheme. This may be worth exploring in the future in an independent and larger PanNET cohort. To the best of our knowledge, this is the first study to demonstrate the prognostic value of miRNA-196a in PanNET supported by long-term survival data.

There are a few recent studies demonstrating the functional role of miRNA-196a. High miRNA-196a levels have been associated growth promoting and antiapoptotic functions,^22^ and miRNA-196a has been shown to promote cell migration and metastasis in gastric cancer cells with radixin as a direct and functional target.^23^ MiRNA-196a has complementarity to homeobox (HOX) clusters and has been demonstrated to repress target HOX genes, including HOXB8, HOXD8, and HOXA7, through posttranscriptional cleavage.^24–26^ In addition, miR-196a has been demonstrated to play crucial roles in proliferation and epithelial-mesenchymal transition in pancreatic adenocarcinomas, possibly by targeting nuclear factor kappa-B inhibitor-α (NFκB1α).^27^ The potential role of miRNA-196a as prognostic and diagnostic biomarkers has been demonstrated in various diseases including familial and sporadic pancreatic cancers and pancreatic intraductal papillary mucinous neoplasm.^20,22,28–31^ For example, serum miRNA-196a levels were demonstrated to be predictive of decreased survival and higher stage of pancreatic ductal adenocarcinomas, and elevated miRNA-196a levels in pancreatic juice samples were found to be predictive of intestinal type intraductal papillary mucinous neoplasm.^30^

Our finding that high miRNA-196a expression was associated with aggressive behavior and poor prognosis in PanNETs, while the screening part of the study revealed decreased miRNA-196a in liver metastases compared to the matched primary PanNETs seems counterintuitive and needs further explanation. The expression of miRNA-196a has not yet been studied in metastatic tumors. Interestingly, miR-27b and miR-142–5p – 2 miRNAs that also showed decreased levels in the liver metastases compared to the primary tumors – were also associated with features of aggressive behavior (higher stages, mitotic counts, Ki-67 labeling indices, and more frequent recurrences) although not statistically significant. In addition, by performing an LNA-FISH analysis on the same 2 matched cases of primary PanNETs and their corresponding liver metastases, we found that miRNA-196a expression was decreased in the metastases compared to the primary tumors (data not shown). Therefore, although functional studies are required for validation, it could be speculated that while miRNA-196a expression is associated with an aggressive behavior of PanNETs, including invasion and metastasis, it may be suppressed once the tumors have metastasized, possibly due to the effect of a new microenvironment.

In conclusion, we demonstrate that tissue miRNA-196a may be a promising prognostic biomarker of recurrence in curatively resected PanNET, although further large-scale studies would be required to incorporate this finding into routine clinical practice.

## Supplementary Material

Supplemental Digital Content
