# Supplementary material for: High Expression of MicroRNA-196a Indicates Poor Prognosis in Resected Pancreatic Neuroendocrine Tumor
Source: Medicine (Baltimore). 2015 Dec 18;94(50):e2224. doi: 10.1097/MD.0000000000002224 (PMC5058906; doi:10.1097/MD.0000000000002224)
Supplement: Supplemental Digital Content [file medi-94-e2224-s001.pdf]

Supplementary Figure 1. LNA-FISH analysis demonstrates increased miRNA-196a signals in a PanNET (A) compared to non-neoplastic endocrine cells (B) and acinar cells (C).

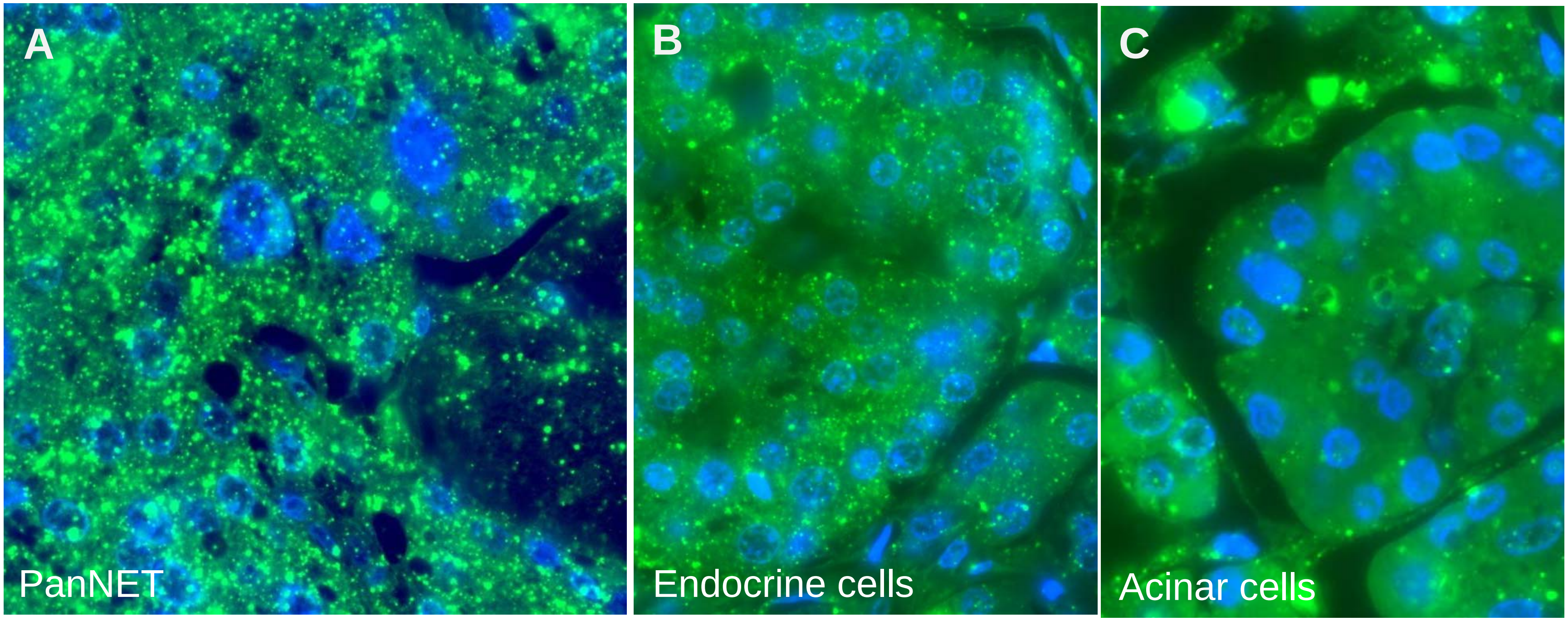

Supplementary Figure 2. Results of ROC analyses. High tumoral miRNA-196a levels significantly discriminated between recurrent and non-recurrent PanNETs with a cut-off level of 1.279 (A). miRNA-27b levels showed similar results but with a lower specificity (B), and miRNA-142–5p levels did not appear to significantly discriminate between recurrent and non-recurrent PanNETs (C).

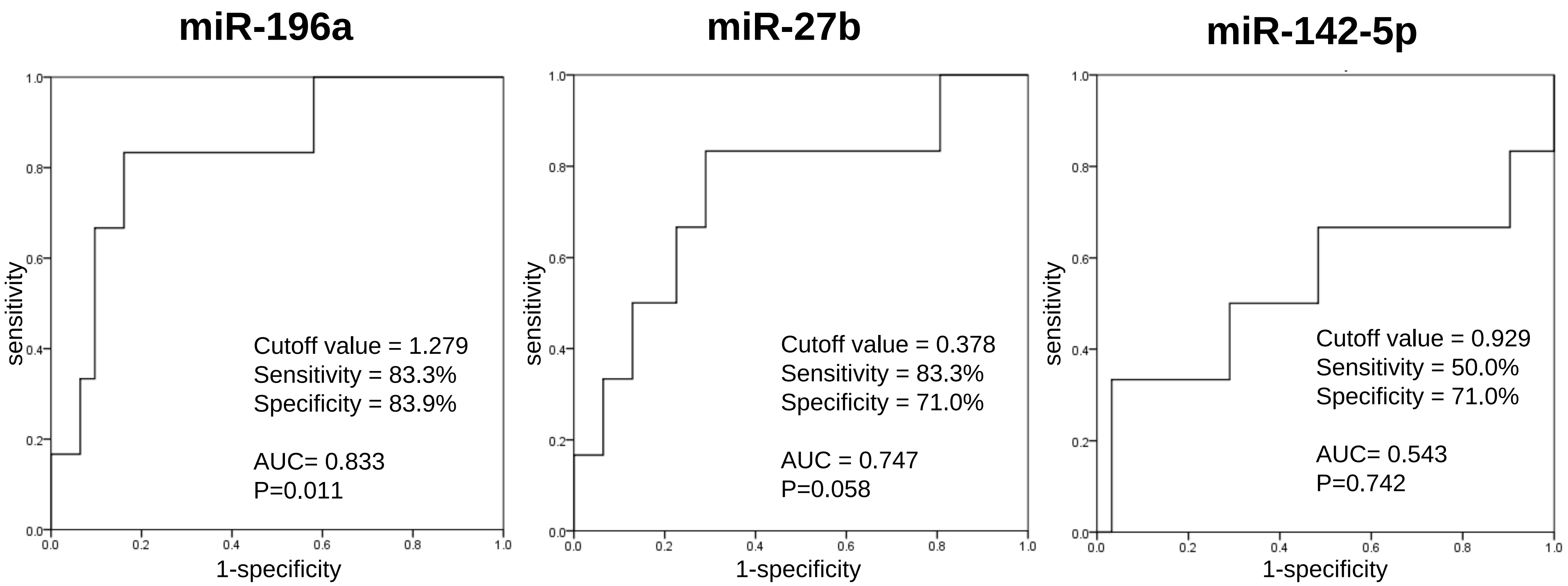

Supplemental Digital Content 3. Cox regression analysis for the recurrence of PanNET

Variable	Crude Hazard Ratio on Univariable Analysis	95% CI	*P*	Adjusted Hazard Ratio on Multivariable Analysis	95% CI	*P*
**miR-27b* (low/high)**	7.637	0.853-68.338	0.069	6.697	0.736-60.903	0.091
**miR-142-5p* (low/high)**	3.189	0.623-16.336	0.164			
**miR-196a* (low/high)**	20.299	2.322-177.413	0.006	16.267	1.732-152.789	0.015

*Cut-off values for miR-27b (0.378), miR-142-5p (0.9285) and miR-196a (1.279) levels were derived from ROC curves.
